# Knowledge translation resources to support the use of quality of life assessment tools for the care of older adults living at home and their family caregivers

**DOI:** 10.1007/s11136-021-03011-z

**Published:** 2021-10-18

**Authors:** Kara Schick-Makaroff, Richard Sawatzky, Lena Cuthbertson, Joakim Öhlén, Autumn Beemer, Dominique Duquette, Mehri Karimi-Dehkordi, Kelli I. Stajduhar, Nitya Suryaprakash, Landa Terblanche, Angela C. Wolff, S. Robin Cohen

**Affiliations:** 1 Faculty of Nursing, University of Alberta, 4-116 Edmonton Clinic Health Academy, 11405-87 Ave, Edmonton, AB T6G 1C9 Canada; 2 School of Nursing, Trinity Western University, 22500 University Drive, Langley, BC V2Y 1Y1 Canada; 3 Centre for Health Evaluation & Outcome Sciences, St. Paul’s Hospital, 588- 1081 Burrard Street, Vancouver, V6Z 1Y6 Canada; 4 Sahlgrenska Academy, University of Gothenburg, Medicinaregatan 3, Box 400, 405 30 Gothenburg, Sweden; 5 Office of Patient Centred Measurement, British Columbia, Ministry of Health, 1190 Hornby Street, 341F, Vancouver, BC V6Z 2K5 Canada; 6 Institute of Health and Care Sciences, and Centre for Person-Centred Care (GPCC), Sahlgrenska Academy, University of Gothenburg, Box 400, 405 30 Gothenburg, Sweden; 7 Palliative Centre, Sahlgrenska University Hospital, Västra Götaland Region, Gothenburg, Sweden; 8 Faculty of Nursing, University of Alberta, Level 3, Edmonton Clinic Health Academy, 11405-87 Ave, Edmonton, AB T6G 1C9 Canada; 9 Division of Geriatric Medicine, Department of Medicine, University of Alberta, 1-198 Clinical Sciences Building, 11350 - 83Avenue, Edmonton, AB T6G 2P4 Canada; 10 School of Nursing, Institute on Aging and Lifelong Health, University of Victoria, STN CSC, PO Box 1700, Victoria, BC V8W 2Y2 Canada; 11 Center for Clinical Epidemiology and Evaluation, University of British Columbia, 7th Floor, 828 West 10th Avenue, Research Pavilion, Vancouver, BC V5Z 1M9 Canada; 12 Departments of Oncology and Medicine, McGill University, Montreal, QC H4A 3T2 Canada; 13 Lady Davis Research Institute of the Jewish General Hospital, Montreal, QC H3T 1E2 Canada

**Keywords:** Quality of life assessment tools, Patient-reported outcome measures, Older adults, Family caregivers, Knowledge translation

## Abstract

**Purpose:**

To support the use of quality of life (QOL) assessment tools for older adults, we developed knowledge translation (KT) resources tailored for four audiences: (1) older adults and their family caregivers (micro), (2) healthcare providers (micro), (3) healthcare managers and leaders (meso), and (4) government leaders and decision-makers (macro). Our objectives were to (1) describe knowledge gaps and resources and (2) develop corresponding tailored KT resources to support use of QOL assessment tools by each of the micro-, meso-, and macro-audiences.

**Methods:**

Data were collected in two phases through semi-structured interviews/focus groups with the four audiences in Canada. Data were analyzed using qualitative description analysis. KT resources were iteratively refined through formative evaluation.

**Results:**

Older adults and family caregivers (*N* = 12) wanted basic knowledge about what “QOL assessment” meant and how it could improve their care. Healthcare providers (*N* = 13) needed practical solutions on how to integrate QOL assessment tools in their practice. Healthcare managers and leaders (*N* = 14) desired information about using patient-reported outcome measures (PROMs) and patient-reported experience measures (PREMs) in healthcare programs and quality improvement. Government leaders and decision-makers (*N* = 11) needed to know how to access, use, and interpret PROM and PREM information for decision-making purposes. Based on these insights and evidence-based sources, we developed KT resources to *introduce* QOL assessment through 8 infographic brochures, 1 whiteboard animation, 1 live-action video, and a webpage.

**Conclusion:**

Our study affirms the need to tailor KT resources on QOL assessment for different audiences. Our KT resources are available: www.healthyqol.com/older-adults.

## Plain English summary

Older adults living at home facing frailty, and their family, often have challenges with quality of life. Tools for assessing quality of life can help make the priorities of patients and families visible to healthcare providers and leaders. But there is a lack of resources to support the use of these quality of life tools by patients, families, healthcare providers, healthcare managers, and government leaders. Our goal was to (1) identify gaps in knowledge, and (2) develop “tailored” resources (e.g., videos, written materials.) to support the use of tools to assess quality of life. We found there was a need for simple, introductory resources to address the knowledge gaps of different audiences. With participants’ input, we developed and tailored resources to introduce quality of life assessment through 8 infographic brochures, 1 whiteboard animation, 1 live-action video, and a webpage. Our findings confirm that different audiences have different needs for resources to support their use of quality of life tools. Our tailored resources are now freely available at www.healthyqol.com/older-adults.

## Introduction

Older adults living at home with frailty often have complex problems that not only affect their ability to function, but also their quality of life (QOL) [1, 2]. Similarly, their family caregivers' QOL can be affected as they often assume primary responsibility for coordinating and providing care, which may create or impact upon their own health issues [3–5]. QOL has been defined by the World Health Organization as “individuals’ perception of their position in life in the context of the culture and value systems in which they live and in relation to their goals, expectations, standards and concerns” [6]. QOL assessments can help to ensure that healthcare for patients and family caregivers is informed by their experiences [7]. QOL assessment tools can facilitate such assessments by asking patients and family caregivers to respond to questions for measuring their QOL, healthcare experiences, physical, mental, and social health. These tools include health-related QOL measures, patient-reported outcome measures (PROMs), or patient-reported experience measures (PREMs). Our review identified 65 PROMs or PREMs used with older adults living at home, and their family caregivers [8]. This project seeks to support the use of QOL assessment tools for older adults by developing introductory and readily available (www.healthyqol.com/older-adults) knowledge translation (KT) resources tailored for different users.

Globally, as the population is aging due to longer life expectancies and decreasing fertility [9], there is increasing requirement for home-based care to support the needs of older adults [10]. In Canada, where our work is located, in 2013, 1.8 million people received publicly-funded homecare, 70% of whom were older adults [11]. There is increasing interest in using QOL assessment tools to support a patient-, person- or people-centered approach to care [12, 13]. These tools are used by different people across micro- (patients, caregivers, and healthcare providers), meso- (healthcare organization managers and leaders), and macro-levels (government) of healthcare [8, 14–17]. At the micro-level, QOL assessments in clinical practice can improve healthcare provider-patient communication, raise awareness of problems that would otherwise be unidentified, improve care plans, and improve multidisciplinary collaboration [18–21]. At the meso-level, healthcare managers and leaders increasingly advocate for the routine use of PROMs and PREMs for patient-/person-centered program evaluation and quality improvement purposes [14, 22]. At the macro-level, use of PROMs and PREMs by government leaders is gaining momentum [23, 24].

Despite decades of research and systematic reviews on using QOL assessment tools, there is a dearth of resources that translate this evidence to support the use of QOL assessment tools by different people across micro-, meso-, and macro-levels of healthcare [25–27]. To support routine use of QOL assessment tools, it is important that all users, including older adults, family caregivers, healthcare providers, and decision-makers, are knowledgeable about what these tools are and how they can be used to improve healthcare. A few resources have been developed to support use of QOL assessment tools, with a primary focus on use by clinicians in practice [28–33]. However, none of the resources focus on contexts of older adult care, nor have they been tailored to address different perspectives and needs of knowledge user audiences at micro-, meso-, or macro-levels [27]. Uptake and evidence-based use of QOL assessment tools requires KT resources be tailored to address the different knowledge needs [34, 35]. This project aimed to address this gap by learning from four audiences about their needs for tailored, evidence-informed KT resources regarding use of QOL assessment tools, including (1) older adults and their family caregivers (micro), (2) healthcare providers (micro), (3) healthcare managers and leaders (meso), and (4) government leaders and decision-makers (macro). Our two objectives were to (1) describe knowledge gaps and resources and (2) develop corresponding tailored KT resources to support use of QOL assessment tools by each of the micro-, meso-, and macro-audiences.

## Methods

### Study design

**Fig. 1:**
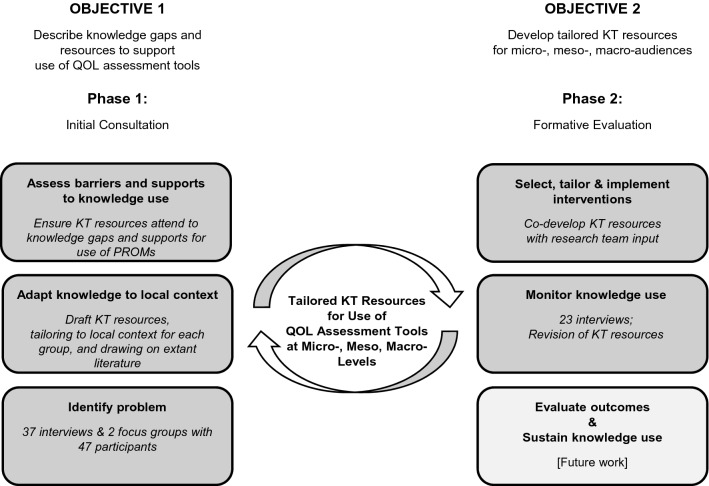
Study Design Guided by Knowledge-to-Action Framework^a^. The Knowledge-to-Action Framework can be regarded as a cycle of integrated knowledge translation (KT), denoted by the circular arrows. In the Initial Consultation (Objective 1/Phase 1), we identified the problem, drafted KT resources for the local context, and ensured they addressed both knowledge gaps and supports. In the Formative Evaluation (Objective 2/Phase 2), we then tailored and revised the KT resources after the 2nd round of interviews with participants. Future work is needed for summative evaluation. ^a^Guided by Graham ID, Logan J, Harrison MB, Straus SE, Tetroe J, Caswell W et al. Lost in knowledge translation: time for a map? *The* *Journal* *of* *Continuing* *Education* *in* *the* *Health* *Professions*. 2006;26(1):13–24. https://doi.org/10.1002/chp.47

Our research was guided by qualitative description methodology [36, 37] and the Knowledge-to-Action Framework [34, 35] (See Fig. 1). There were two phases: (1) “Initial Consultation” involved recruitment, data collection, and analysis focused on objective 1 as the basis for initial development of KT resources using input from micro-, meso-, and macro-audiences and drawing from evidence-based resources regarding the use of QOL assessment tools. (2) “Formative Evaluation” involved ongoing refinement and iterative evaluation of the KT resources with study participants (objective 2). Our KT team was established to ensure representation of different audiences by including clinicians, healthcare leaders, patients, family caregivers, members from patient advocacy groups and non-profit organizations, and researchers with expertise in QOL assessment and person-centered care.

### Settings and sample

We created the following four participant groups at micro-, meso-, and macro-levels sampled from healthcare across Canada: (1) adults ≥ 65 years old, receiving home care for chronic conditions associated with frailty [1, 2] (e.g., cancer, cardiac, respiratory, endocrine, musculoskeletal conditions, and co-morbidities), and their family caregivers (micro); (2) healthcare providers of older adults living at home (micro); (3) healthcare managers and leaders responsible for providing care to older adults (meso); and (4) provincial government leaders and decision-makers of healthcare organizations (macro) responsible for home health services for older adults.

We used purposive and snowball sampling to obtain a diversity of perspectives (albeit limited to people conversant in English) by inviting older adults and family caregivers with different health conditions, genders, and other participants with various roles and responsibilities. All patients, caregivers, and many healthcare providers (micro) were recruited via telephone or e-mail from an ongoing project on QOL assessments in home care for older adults [38]. Within that project, they had experience with an electronic QOL assessment and practice support system (“QPSS”). Healthcare managers and leaders (meso), as well as government leaders and decision-makers (macro) were recruited via KT team members’ networks, and invited by e-mail. Twenty-seven people declined participation; no one withdrew.

### Data collection

Data were collected via 37 interviews and 2 focus groups for the Initial Consultation phase (Nov. 2018–Mar. 2019), and via 23 interviews for the Formative Evaluation phase (Aug. 2019–Oct. 2019). Interviews/focus groups were conducted by the study co-lead (KSM) and five trainees (one nurse, three nursing students, one anthropologist), in homes (*n* = 20, with older adults or family caregivers), at workplaces (*n* = 7), or on the phone (*n* = 35), lasting 45–90 min. There were no prior relationships between interviewers and participants. Debriefing meetings between interviewers and study leads facilitated trainee interview training, ensured data quality, enhanced reflexivity, and oriented the interviewers to the data.

We developed semi-structured interview/focus group guides with open-ended questions for each participant group and the project’s two phases. During the Initial Consultation, we asked participants to first discuss their experience and familiarity with QOL assessment tools, and then more specifically to discuss knowledge gaps, opportunities, and challenges regarding use of QOL assessment tools, as well as supports, benefits, and recommendations to facilitate their use of QOL assessment tools. For the Formative Evaluation, we invited participants to review and provide feedback on the KT resources. The guides went through iterative rounds of development with the KT team. Interviews/focus groups were recorded and transcribed.

### Data analysis methods for objective 1

Guided by qualitative description analysis [36, 37], data were coded and categorized to describe knowledge gaps and resources to support the use of QOL assessment tools. Rather than define knowledge gaps as deficits, we viewed them as “a fruitful site for the development of knowledge and practice capacity” (p. 179) [39]. Supportive resources were considered in relation to self (micro), patient-care (micro), program delivery/quality improvement (meso), or policy development (macro), and included recommendations for tailored KT resource development.

The transcripts from the first interviews were re-read to generate the initial codebook, which was iteratively refined to arrive at categories of knowledge gaps and supportive resources for the use of QOL assessment tools. N-Vivo^tm^ software was used to facilitate collaborative coding. Data from the four participant groups were analyzed separately, and then compared, linked, and contrasted [40] to inform the development of tailored KT resources. One experienced qualitative analyst and two trainees coded the data under the supervision of the lead researchers (RS, KSM). Differences in coding were discussed with trainees and supervisors, and decisions were made through dialogue and consensus. Saturation was achieved [41, 42] when no new categories were identified and when participants’ descriptions became repetitive.

### Data analysis methods for objective 2

We followed integrated-KT principles [34, 35] to design and tailor KT resources for each participant group using learnings from the Initial Consultation while drawing upon evidence-based sources about using QOL assessment tools. The KT resources were collaboratively developed through several rounds of revision to keep the resources consistent, easy to read (for the intended audience), and relevant. Our KT team worked with a content editor to ensure consistency of language and messages and a graphic designer to create a simple, harmonious design across all resources.

The KT resources were refined through a formative evaluation process [43] based on participant feedback obtained via the interviews. Before the interviews, participants viewed the KT resources through various means: online, through a recorded webinar, or on a tablet with a KT team member prior to the interview. Participants shared their perspectives and provided input to ensure the resources were easy to understand, appealing, relevant, appropriate, and facilitated understanding of QOL assessments. The same qualitative description analysis methods as for objective 1 were followed to describe the participant’s evaluative feedback to tailor KT resources for micro-, meso-, macro-audiences.

### Rigor

To attend to trustworthiness, we drew upon qualitative principles of credibility, transferability, and confirmability [40, 44]. Credibility was upheld through longitudinal engagement, iterative cycles of engagement, and member-checking with the four groups of participants and members of our KT team. Field notes written after each interview included reporting of context, and interviews were discussed during team meetings. Confirmability was established through a detailed audit trail ensuring that records were kept of design and analytic decisions. Preliminary analysis and category development were presented to the full KT team on 6 occasions through video conference or email, and their feedback was incorporated into the refinement of both the codebook and ongoing analysis. These team discussions facilitated reflexivity and were critical to the integrity of the project. The Consolidated Criteria for Reporting Qualitative Research guidelines were followed [45].

## Results

### Phase 1: Description of knowledge gaps and supportive resources

Forty-seven participants consented to participate including 7 patients, 4 family caregivers, 13 healthcare providers, 14 healthcare managers and leaders, and 11 government leaders and decision-makers (Table 1). Participants in all groups were predominantly female and of European or Canadian descent.

**Table 1:** Phase 1 (initial consultation) and phase 2^a^ (formative evaluation) study participants

Demographic information	Older adults	Caregivers	Healthcare providers	Healthcare leaders	Government leaders	Total
Total unique participants	7	5	13	14	11	50
Phase 1 (Phase 2)	7 (4)	4 (5)	13 (4)	14 (6)	9 (4)	47 (23)
Province of residence						
British Columbia	3 (2)	2 (3)	6 (1)	11 (5)	2 (2)	24 (13)
Alberta	4 (2)	2 (2)	7 (3)	1 (1)	2 (0)	16 (8)
Other	0 (0)	0 (0)	0 (0)	2 (0)	5 (2)	7 (2)
Gender						
Female	6 (3)	4 (4)	9 (2)	13 (6)	5 (3)	37 (18)
Male	1 (1)	0 (1)	4 (2)	1 (0)	4 (1)	10 (5)
Age						
Mean age (years)	84 (85)	69 (67)	46 (56)	53 (54)	47 (52)	
Range (min–max)	67–93	65–77	33–63	37–65	31–58	
Marital status						
Married	1 (1)	4 (5)	N/A	N/A	N/A	
Widowed	4 (2)	0 (0)	N/A	N/A	N/A	
Never married	2 (1)	0 (0)	N/A	N/A	N/A	
Highest level of education						
High school	1 (0)	0 (0)	0 (0)	0 (0)	0 (0)	1 (0)
College, trade, or CEGEP degree	4 (2)	0 (0)	0 (0)	0 (0)	0 (0)	4 (2)
University Undergraduate	1 (1)	1 (1)	4 (0)	1 (1)	0 (0)	7 (4)
University Master’s	1 (1)	3 (4)	7 (4)	9 (3)	6 (2)	26 (13)
University Doctoral/Medical	0 (0)	0 (0)	2 (0)	4 (2)	3 (2)	9 (4)
Employment status						
Part time work	0 (0)	0 (0)	0 (0)	2 (1)	0 (0)	2 (1)
Full time work	0 (0)	1 (1)	13 (4)	12 (5)	9 (4)	35 (14)
Retired	7 (4)	3 (4)	0 (0)	0 (0)	0 (0)	10 (8)
Range in current position (years)	N/A	N/A	1–29	1–15	1–18	
Annual salary						
< $31,000	1 (1)	0 (0)	N/A	N/A	N/A	
$31,000 to $50,000	5 (3)	1 (1)	N/A	N/A	N/A	
$50,000 to $70,000	1 (0)	0 (0)	N/A	N/A	N/A	
$70,000 to $90,000	0 (0)	1 (1)	N/A	N/A	N/A	
> $90,000	0 (0)	2 (3)	N/A	N/A	N/A	
Self-reported ethnic background^b^						
Canadian	2 (0)	0 (0)	1 (1)	N/A	N/A	
Caucasian	0 (0)	1 (2)	6 (2)	N/A	N/A	
European decent (English, English/Scottish/Irish, German, Scottish/French, Ango-Saxon, Scottish/British, Irish/Scottish, European)	5 (4)	2 (2)	2 (0)	N/A	N/A	
Japanese Canadian	0 (0)	1 (1)	0 (0)	N/A	N/A	
Indo-Canadian, Asian, Pilipino	0 (0)	0 (0)	3 (1)	N/A	N/A	
Iranian	0 (0)	0 (0)	1 (0)	N/A	N/A	
Born in Canada	5 (3)	4 (5)	11 (4)	N/A	N/A	

^a^Phase 2 is denoted in round brackets. Note that there were 3 participants in Phase 2 who were not a part of Phase 1 (1 caregiver and 2 government leaders), and 26 participants who were in Phase 1 but not a part of Phase 2

^b^Participants self-reported their ethnic background. All narrative responses are described here verbatim

N/A indicates that the information is not collected. Information about ethnicity was not obtained from healthcare leaders or governments as they constitute a very small sample of public officials who may be identified. Information about marital status and salary of healthcare providers, leaders, and government was not obtained because it was not required for analysis

The Initial Consultation facilitated description of the knowledge gaps and resources to support use of QOL assessment tools. While knowledge gaps were primarily related to barriers to knowledge use, these were interconnected with pragmatic, organizational, or systems barriers experienced by participant groups. Findings from the Initial Consultation informed preliminary development of tailored KT resources. See Table 2 for details with objectives, categories, and exemplar quotes.

**Table 2:** Initial consultation—exemplar quotes

Older adults and caregivers		
Objectives (mapped onto interview questions)	Categories	Exemplar Quotes
Knowledge gaps (barriers) to using QOL assessment tools (“Please share with me your experience using QOL assessment tools?”)	• What does quality of life assessment mean? • What is the objective of these assessments? • Who is filling out the assessments? • How is the data going to be utilized? • How are the tools going to improve my care? • How is the data going to accurately represent my quality of life?	“You’ll have to refresh my memory because I’m not quite sure what you mean by quality of life assessment”PAC#1 “I understand them, but I don’t know how helpful they would be, no matter what you put down there. Say for example like you ask me, “Over the past two days, my life was utterly meaningless and without purpose,” zero to 10. I mean if you pick the zero, I presume you’re ready to jump in the river, right? But a 10, what would that tell you? What are you going to do with information like that?” PAC#10
Supports to using QOL assessment tools: Perceived benefits (“I’d like to know about any ways in which QPSS assessments has been helpful to you and your care, and ways it could be more helpful in your care?”)	• Helpful in self-care • Helpful to other patients • Helpful to the system in making changes and improvements	“Well, every time you’re asked questions about activities, personal activities, maintaining health… like, health maintenance is so important at an advanced age, and every time you’re reminded of something, I think it encourages you to keep on doing whatever health activities you’ve been told you must maintain. I think that the more seniors can be reminded that they’re not alone, there’s a whole lot of us out there, the better it is, and the more encouraged we are to participate in our own healthcare maintenance." PAC#14
Supports to using QOL assessment tools: Recommendations of resources (“Are there any resources that might be useful to you or other patients/caregivers or that would help you and other patients/caregivers complete these QOL assessments routinely either by yourself or with your healthcare provider?”)	• Material (pamphlet or videos, written blurbs, read-aloud surveys) • Services (routine training, active one-on-one or group engagement sessions)	“Well, whatever information it is that you’re trying to present, you would want people to be able to see it, to hear it well, and to come away from it feeling that they had new information, would help not only themselves but other people, maybe in their family or down the street.” PAC#14 “Yeah, it might be helpful to have a video tutorial. I don’t think, for most people, having more written material is the way most people want to go. But it’s the kind of thing I’m sure that if you said, “We had a two-minute video” or “a five-minute video” or something, I may watch. If you sent me a package of material that’s 20 pages long, I’m probably not going to read it, or at least all of it or with a great deal of attention.” PAC#3

Healthcare providers		
Objectives (mapped onto interview questions)	Categories	Exemplar Quotes
Knowledge gaps (barriers) to using QOL assessment tools (“Please share with me your experiences with using QOL assessment tools or using the Quality of Life and Practice Support System in your practice?”)	• How to use it in our practice? • Who is going to initiate the assessment? • How is success going to be measured? • How are different case loads being considered? • How are caregivers being supported?	“I think the knowledge piece… I think a few things. There’s the lack of recognition of the tool, the awareness that the tool exists and how to access it, where it’s found in the chart, how to get that data, and the validity of the data. And really the question is, how will clinicians be able to process this information in a way that kind of makes it fun, makes them think they’re really changing up how they practice, but that doesn’t add workload?” HCP#4
Supports to using QOL assessment tools: Perceived benefits (“How has the electronic QPSS system or paper QOL assessment tools been helpful to you in providing care to the frail elderly and their caregivers?”)	• Improves healthcare providers’ critical thinking and reasoning to provide better patient- and family-centred care	“We can see the trends and we can just discuss ‘Okay, well, what’s changed compared to last month or a couple of months ago?’ We could use it as a case study, so we can see what’s happening on a specific client, you know, the graph was here and now the graph is down here, and that would be kind of interesting to see, why is this… what’s happening with this client and the family and bringing that up. I think it improves our critical thinking skills and coming together as a team, it will help us, each other, and for the client and family as well.” FG#1 P1
Supports to using QOL assessment tools: recommendations of resources “Thinking about your experiences using *QOL* assessment tools in your care, can you recommend any resources, like educational material for example, that would be especially beneficial in helping you routinely use these tools?”	• Material (electronic and paper guidelines /quick tips/checklist; educational resources; online learning modules; a resource directory/referral list/resource tree; infographics) • Services (training and mentoring; continuing education; alarm or trigger notification; system integration)	“The quick tips, because I know P3 has made up these little quick-tip sheets for us that make it… you can sort of access it and what exactly are you looking. Anything that can help you manage a system or do a process… Checklists. Like a quick checklist. it just makes it easier and quicker … kind of like a quick overview… Because a lot of times when you’re sitting and you have multiple systems open on your desktop and then I have this piece of paper here that I just kind of flip through, and I just kind of go through and make sure that I have everything I need before I save and complete.” FG #1 P2

Healthcare managers and leaders		
Objectives (mapped onto interview questions)	Categories	Exemplar Q uotes
Knowledge gaps (barriers) to using QOL assessment tools (What concerns might you, or other people in your organization or program have or foresee regarding the use of PROMs and PREMs?”)	• How to operationalize deploying PROMs and PREMs in the real world? • How to use PREMs information to change clinical practice? • What are the similarities and differences between QoL information and clinician’s assessments? • What are the financial implications of using QoL information in decision-making at the organizational level?	“How does that (QPSS tools) help support the journey of the patient to get to where they feel or how they define health and wellness for themselves? How are we empowering the patients to understand what this data is and what we could use it for, and being able to speak to it and bring it to the conversation? How are we thinking provincially on a bigger scale? What are we actually using this information for? How are we then translating that down to the people who need what information?” HML#15 “Would this be a completely separate system? How would it interact with the current health authority systems? Are there any confidentiality and privacy issues with that?” HML#16
Supports to using QOL assessment tools: Perceived benefits (“Please share the successes if any, your organization or program have had regarding the use of PROMs and PREMs.”)	• Helpful in providing better quality care/service • Ensuring client’s needs are met • Helpful as a decision-making tool	“All of that data that the patient is pushing to the home care nurse enables them to make different decisions. The other thing it does is it enables them to go more deeply into assessment on the call. If you have the reported outcomes being pushed to the nurse, who then can view them, then she or he can do more deeply in, and not spend all of that time in screening.” HML#9
Supports to using QOL assessment tools: Recommendations of resources (“Based on your experience using PROMs and PREMs, please share with me any resources, like clinical pathways, guidelines, forums, list serves etc. that would help leaders integrate PROMs and PREMs in their organization or program?”)	• Material (short online modules and webinars; journal article, lunch and learn; PDF cycles; clinical education, right assessment tool) • Services (quality of life coach; helpline; interpreter; communities of practice; user groups; Task group with weekly Conferences-Brief Action Planning tool; incentives like accreditation)	“I think multiple mediums is always a good thing, clinical support tools, maybe a learning module, a video. You know, a YouTube video. I think that the practical application piece you can only do through some sort of media medium. Because I find that a lot of the nurses and interprofessional team really prefer being able to access educational information any time of the day or night. So being able to have those platforms of access to educational resources to me would be the best way to go when we talk about continuing education. And that is current, right? So, it’s current, it’s evidence-informed.” HML#42

Government leaders and decision-makers		
Themes	Categories	Exemplar Quotes
Knowledge gaps (barriers) to using QOL assessment tools (“What concerns might you, or other people in your organization have and what challenge do you foresee regarding the use of PROMs and PREMs/QOL assessments informing healthcare decision-making or healthcare policy?”)	• How the data collected could be useful in driving quality improvement initiatives • How the assessment data can be used at the macro-level to inform decision-making	“I think the challenge for us is, how do we collect the data? And then, if we collect the data, is it for system planning or is it for individual patient feedback? And it’s a challenge to collect for both, because probably the way you collect it might be different and so it is sort of a question of, what are we going to use the data for? And then, how do we collect it routinely? How does it get used?” GML#2 “I think that they’re [leaders] well aware of the importance of including patient voice, even if they’re not sort of aware of quality of life assessment tools. But I’m not sure that they have the knowledge to work with that information at this point and to… you know, they just haven’t been taught that this is a good way of making decisions or contributing to decisions.” GML#5
Supports to using QOL assessment tools: Perceived benefits (“What do you think is the value of collecting patient perspectives to guide and inform complex policy decisions?”)	• In resource allocation decisions in partnership with patients • Understand trends and relationships for policy and evaluation • Improving quality, service, and patient outcomes	“The dream for me is that it is useful for being able to direct resources to those areas that patients are telling us is most needed, rather than something for clinicians or administrators to think they know where the resources should go. But the dream is that we’re making these kinds of decisions not just from behind the desk but in partnership with patients. That’s what PREMs and PROMs are all about is making sure the patients are included. So, that’s the dream, whether or not it’s a realistic reality.” GML#5
Supports to using QOL assessment tools: Recommendations of resources (“Are there any recommendations or resources that you need or that might be useful to you or other government leaders to use PROMs and PREMs for health policy at regional, provincial or national levels?”)	• Material (case studies demonstrating relevance or resources; educational sessions where people manipulate their own data; stories and visualizations; face to face sit-downs with providers and policy makers; webinars, workshops, educational sessions, interactive online modules; toolkits; interactive database; sharing information through journals short videos.) • Services (in-house internal data manager or QI specialist; research support unit like the APERSU (Alberta PROMs and EQ-5D Research and Support Unit)	“I think the best resources are when the data is presented in using data visualization where you can see by looking… it’s intuitive when you look at the picture or the infographic or whatever it is. It’s about the data display in a way that people can understand it without necessarily understanding all the methodologies and statistics that sit underneath it. So, data display techniques that are clear and intuitive, I think those are the best kind of resources. The other thing that we talk a lot about is storytelling as being effective means to communicate, right? So, what’s the story, and what’s the patient’s story and the individual that might, you know, shed light on what the data means?” GML#1 “I think like brief toolkit, like brochure-ish type explanations, graphic examples, little videos. I think those would be… Definitely demonstrations of actual use, like vignettes of how the systems can be… how quality of life information can be incorporated into decisions, like with real examples” GML#7

Each of the four participant groups had unique needs regarding use of QOL assessment tools. Older adults and family caregivers desired basic knowledge to improve their understanding of what “QOL assessment” meant and how information about their QOL could be used to improve care. They wanted to know how their responses could actively be used to inform their self-care or the healthcare they received. As one older adult said, *What* *are* *you* *going* *to* *do* *with* *information* *like* *that?* They recommended that resources could include videos or pamphlets, and that these resources address how to answer questions in QOL assessment tools.

Healthcare providers wanted practical, didactic information about how to implement QOL assessment tools without adding to their workload, and how to use QOL information to inform care decisions with patients and caregivers. Providers stressed the importance of integrating the QOL assessments within existing work structures and health records. One said, *How* *will* *clinicians* *be* *able* *to* *process* *this* *information* *in* *a* *way* *that* *kind* *of* *makes* *it* *fun,* *makes* *them* *think* *they’re* *really* *changing-up* *how* *they* *practice,* *but* *that* *doesn’t* *add* *workload?* Healthcare providers recommended that KT resources should sustain their interest, be concise, provide examples, and pragmatically support their use of QOL assessments in practice regardless of the mode (i.e., electronic or paper).

At meso- and macro-levels, healthcare and government leader participants used the terms PROMs and PREMS to refer to QOL assessment tools. Healthcare managers and leaders recommended that KT resources show them how PROMs and PREMs could be used in organizational decision-making at a meso-level. They needed information to understand how these tools could be used in their practice overseeing healthcare programs, including quality improvement. One leader asked, *How* *are* *we* *thinking* *provincially* *on* *a* *bigger* *scale?* *What* *are* *we* *actually* *using* *this* *information* *for?* *How* *are* *we* *then* *translating* *that* *down* *to* *the* *people* *who* *need* *what* *information?* They also felt constrained by a lack of time, lack of training, and other structural barriers related to integration with electronic health systems. They specifically recommended resources on implementation of QOL assessment tools and interpretation of results.

Government leaders and decision-makers needed knowledge about how to access, use, and interpret PROM and PREM information in their decision-making. One government leader said, *The* *dream* *for* *me* *is* *that* *it* *is* *useful* *for* *being* *able* *to* *direct* *resources* *to* *those* *areas* *that* *patients* *are* *telling* *us* *is* *most* *needed,* *rather* *than* *something* *for* *clinicians* *or* *administrators* *to* *think* *they* *know* *where* *the* *resources* *should* *go.* *But* *the* *dream* *is* *that* *we’re* *making* *these* *kinds* *of* *decisions* *not* *just* *from* *behind* *the* *desk* *but* *in* *partnership* *with* *patients.* They expressed concern regarding the use of different tools across organizations, costs or resources, and time-lags between collection and reporting. Government leaders and decision-makers recommended that the KT resources offer examples of use in decision-making, support interpretation of data, be concise but also come alive through stories, and be tailored to their scope of practice.

### Phase 2: Development of tailored KT resources

Twenty-three participants took part in the Formative Evaluation including 4 patients, 5 family caregivers, 4 healthcare providers, 6 healthcare managers and leaders, and 4 government leaders and decision-makers. All but 3 of them also participated in the Initial Consultation. (See Table 1 for participant characteristics.)

#### Description of initial KT resources

There was a need for simple, introductory didactic resources on QOL assessments tailored specifically to the context and needs for each group. In various ways, each group asked, “what’s in it for me?” Table 3 outlines the KT resources that addressed this question, developed collaboratively with members of our KT team, while drawing on evidence-based sources.

**Table 3:** Knowledge translation resources

Participant group	KT resource*	Description
Older adults and family caregivers	Brochure: "Live your Best Life Possible"	Provides a general overview about QOL assessments
	Brochure: “Frequently Asked Questions (FAQs) about QOL Assessments”	Addresses questions that older adults and family caregivers asked, with responses informed by evidence-based sources
Healthcare providers	Brochure: “Start the Conversation about QOL Assessments”	"Conversation starters" for clinicians to introduce QOL assessment tools, along with talking points to use in their interactions with older adults and family caregivers
	Brochure: “The Truth about QOL Assessments”	An infographic that addresses possible misconceptions about QOL assessment tools, as well as evidence-informed responses to address them
Healthcare managers and leaders	Brochure: “Start the Conversation about QOL Assessments”	Provides talking points for healthcare managers and leaders when speaking with other leaders or decision-makers
	Brochure: “Making a Difference with QOL Assessments”	A fact sheet that offers statistics and details about QOL assessment tools for older adults and family caregivers
Government leaders and decision-makers	Brochure: “Fact Sheet about QOL Assessments”	Provides an overview of what and how QOL assessment data can be used to inform decision-making, and offers evidence about the value of QOL assessments
	Brochure: “Frequently Asked Questions (FAQs) about QOL Assessments”	Addresses questions that decision-makers asked about use of data to inform decision-making, with responses informed by evidence-based sources
All four participant groups	Whiteboard: “A Better Life: QOL Assessments”	Whiteboard style animation introducing QOL assessment tools and their use in decision-making at the micro level of healthcare
	Video: “A Better Life”	Live-action video describing the use of QOL assessments in clinical practice and its impact on older adults and family caregivers
	Additional Resources and Supporting Evidence	Additional resources include a full references list for all KT resources, an environmental scan of available resources, and acknowledgements
	Webpage: www.healthyqol.com/older-adults “QOL Assessments for Older Family Caregivers”	Webpage that provides an introduction and access to each of the above resources

*Each resource is available at https://www.healthyqol.com//older-adults/older-adults. References for substantiating each statement in each KT resource are provided in a separate document. See: https://www.healthyqol.com/files/Quality-of-Life-Resources-Citations.pdf

Brochures were tailored to address the knowledge gaps of each audience. For older adults and family caregivers, brochures offered information about the use of QOL assessment tools to live their *best* *life* *possible.* Brochures for healthcare providers were designed not only to help them incorporate QOL assessments into their interactions with older adults and family caregivers, but also to address common myths and misconceptions about the use of QOL assessment tools. For healthcare managers and leaders, the brochures were designed to support discussions about the importance and use of QOL assessments in their organizations for person-centered care and quality improvement. The brochures for government leaders and decision-makers included a “fact-sheet” and “frequently asked questions” focused on the use of evidence-based QOL assessment tools and data to monitor performance, improve quality, and make policy and budget decisions regarding healthcare for older adults and their family caregivers.

Several resources were created that spanned participant groups. One whiteboard animation (2 min) introduced QOL assessment tools and use of this information in micro-level decision-making. A live-action video (6 min) depicted a case study of a father and a son who is a caregiver, using QOL assessment tools to inform their relationship and improve decision-making about care. Statements in each KT resource were referenced with evidence-based sources.

#### Formative evaluation to refine KT resources

Formative evaluation of the KT resources identified an overall positive response by all four participant groups, and all offered suggestions for refinement. See Table 4 for participants’ comments, suggested changes (with exemplar quotes) to each KT resource, and revisions made.

**Table 4:** Formative evaluation—exemplar quotes

Older adults and family caregivers		
Exemplar quotes by participants: overall responses to the resource	Exemplar quotes by participants: suggested revisions	Revisions made to the KT resource
Brochure: “Live your Best Life Possible”		
“Oh, from my perspective, this is fine because it tells you what you need. I think I sort of got the picture, so I don’t think I have any particular misunderstandings about what they’re for. So, if this were first exposure, it’s reasonably clear. So, better understand your health, better understand… Yeah, so it’s there, and readers are going to read it, and they’re going to get it. These tools support quality of life assessments. They consist of simple-to-answer questions about your health, your life, your care. I like the repeating of the phrase ‘living your best life possible,’ because some days that doesn’t look very good for a lot of people.” PAC#15	“I’m trying to put myself in the shoes, the moccasins of somebody who has a Grade 8 reading level. I think it’s probably still pretty good, but for instance, ‘healthcare team,’ what does that mean because that term is used several times on this pamphlet, and I don’t know what that means to the average home care client.” PAC#9	Made some minor edits that included adding an extra letter spacing and different font in tabs; Changed title above Venn diagram to "Quality of Life Assessments"; Changed sentence under "Proven Healthcare Tools Can Help" to "Tools for Quality of Life Assessments"; Added "Assessment" to title of sample questions; Changed "team" to “providers” in multiple places
Brochure: “Frequently Asked Questions (FAQs) about QOL Assessments”		
“I don’t think the average person knows terribly much (about QOL assessments) unless somebody directly says that to them. I certainly hope this resource would probably get them thinking about some of those things a little bit." PAC#15	“I’m sorry, my reaction is, ‘Frequently asked questions about quality of life…’ they’re not questions about quality of life. They’re about quality of life assessments. The emphasis needs to be on the fact that this an FAQ about assessments, a how-to on assessments or a why-to, actually… It’s more like a why-to on assessments, rather than anything else. Not about quality of life- very confusing if you keep intermixing those terms.” PAC#15 “Well, what is meant by ‘support system’? I don’t think you’re talking about people’s walkers there…?” PAC# 9	Edited brochure to include extra letter spacing and different font in tabs; Added "Assessments" to title; Changed "support system" to "social support"; Combined first 2 bullets under "Your answers can help you"; Corrected grammatical word errors

Healthcare providers		
Exemplar quotes by participants: overall responses to the resource	Exemplar quotes by participants: suggested revisions	Revisions made to the KT resource
Brochure: “Start the Conversation about QOL Assessments”		
“I think the visual that you have – sorry, that cloud or whatever – I think that that nicely outlines, start the conversation because you relate that type of symbol or that drawing to a discussion. I think that just the way that’s set up on the left-hand side, I’m assuming you’re trying to make it look like it flows from one – you know, from professionals down to family – and then down to client and then down to… like, client, families, and then down to the assessment. So, I think the flow is good. It’s not focusing just on the client, but it’s also pulling in the family caregiver or the primary caregiver, which I think is really important if we’re going to be looking at a sort of patient-centred, family-centred care.” HCP#9	“There’s too many words. When you’re targeting healthcare providers, less is more because otherwise you just get the ‘zzz.’ They’re just not going to read it. So, you could leave out that, ‘Ask older adults living with chronic condition or their family care…’ You just say, ‘Quality of life assessments include a series of questions about their viewpoint, their health, what matters to them in their healthcare experiences.’ Less is more, and condensing it more.” HCP#3	Shortened description in the title, and corrected "populations" and "groups" to “population” and “people”
Brochure: “The Truth about QOL Assessments”		
“It was just reassuring because it sounds like, you know, sometimes you feel that if you’re giving too many questionnaires, that people feel like you’re not relating to them personally. But it sounds like it’s the opposite that it can enhance your professional relationship because they understand that you care about them and want to find out more. … it seems like there’s no lose and it’s a win/win. So if anything, it can just enhance the information that you already think you know about them and help you deliver better care.” HCP#10	“I thought it would be nice, and perhaps it’s just myself, but I’d love to see some references to support the statements – myths and/or fact – even just as a footnote on the bottom so if anyone was interested they’d be able to see where it was coming from.” HCP#8	Made some minor edits for consistency across the material; Moved the description under the title; Used abbreviated QOL; Included tagline “Learn more at: healthyqol.com and find additional resources and supporting evidence”

Healthcare managers and leaders		
Exemplar quotes by participants: overall responses to the resource	Exemplar quotes by participants: suggested revisions	Revisions made to the KT resource
Brochure: “Start the Conversation about QOL Assessments”		
“I like it. I think that it’s to the point, it’s clean. It has information that will be good to start the conversation if we were to start a conversation with staff. The language, I think it’s perfect. I really like it. I think this is going to be a good resource. If you were asking me to approve it, I would approve it as it is.” HML#6 “Having some tools and resources that you would be able to take to discussions in order to be able to facilitate why it’s important to have a focus on this from a policy and a practice perspective is helpful. And it does I think help to guide some of the key points. I think it’s also good, particularly when you’re using it from a strategic perspective, to have the individuals who are going to be taking this forward have some consistent key messages” HML#11	“This tool, the infographic tool, is part of it for home health in the context of populations you’ve talked about, as well as for older adults, to give them some information? So, frailty is not defined anywhere, at least that I could… I couldn’t find it. I would re-sequence the introduction, and I would first start with the needs of the older adults versus it being first about the home care performance and accountability, so that the driver always becomes about the older adults and it’s very client-centred versus it being about performance and accountability.” HML-042 There’s just too much information, too many words. That there are talking points. Yeah, I think they could be ‘conciser.’ It’s quite a bit of narrative. Managers and leaders, what I find is the more that you go up the organization, the simpler things have to get because the – what do you call it – the bandwidth is very narrow. So, having a seven-line paragraph is too much.” HML#9 “So, for a leader, everything should be meaningful, right? So, talking point one, well, what is that about? What is the stuff underneath about? I think you could take up the talking point one, two, and three and have those provide a very concise bit about what the talking point is about.” HML#9	Provided more clarity throughout the document; Added the word "Assessments" in the title; Bolded and italicized "family caregiver" and "older adults" in the title; Broke up the information into shorter bullet points instead of a long paragraph; Removed word titles under "Talking Point” and replaced with numbers; Switched the order of numbers; Made #3 into two paragraphs; Quotes put in bold purple for better contrast
Brochure: “Making a Difference with QOL Assessments”		
“I thought it did a great job, I can see both at the patient level and the benefits at the population level why would it be beneficial, because I think sometimes when you’re down more on the ground level, like me as a physician, I could argue I could get the information maybe in a different way than a standardized tool but then to say, “Okay, but when we’re trying to take that up to the next level of the population,” and the value of that, I thought that made that very clear.” HML#10	“Like I have short hair, so I don’t necessarily think that this is a man or this is a woman, or that they are only men. This is my thing. Well, the only thing that it could be different maybe, it is to have some colour on these people, as they are all-white. So, have like different colours maybe. This could be something. But only that.” HML#6 “If we had another tab that would have literature about quality of life assessments in general, like the importance, all those things that you ended up putting into this. So, I would like to see some papers here, like to have them available for me to look at if I wanted.” HML#6	Moved sentence "this resource focuses on…" to the introduction; Bolded "older adults" and "family caregivers" in the introduction; Deleted the line between individual and population level; Revised #1 under population level to read, "optimize quality improvement initiatives to better meet the needs of older adults and their family caregivers" instead of "optimize healthcare system performance and quality today and over time"; Included a line at the bottom of this resource “Learn more at: healthyqol.com and find additional resources and supporting evidence” to steer readers towards the references

Government leaders and decision-makers		
Exemplar quotes by participants: overall responses to the resource	Exemplar quotes by participants: suggested revisions	Revisions made to the KT resource
Brochure: “Fact Sheet about QOL Assessments”		
“I think the fact sheet is… you know, it’s clear. It’s concise. It avoids, for the most part, healthcare jargon. Again, it’s because I would see this potentially being a tool that could be used more broadly than in healthcare. So, I think it, for the most part, avoids some of the healthcare jargon that we very easily get tied into, I think the potential here is that this could be quite useful. It’s a good look. It’s clean. It’s eye-catching. I think colour is used appropriately. Text quality is good. I like the graphic. ‘Together, let’s build a person-centred healthcare system.’ I like the graphic. I think they can broadly apply across government so that they can utilize it in their planning and thinking about their service delivery models.” GML#9	“…the only thing I don’t like about this actually is this first paragraph, ‘these tools are for all persons that live with health challenges, including caregivers.’ I find that wording to be a little awkward. Like it’s not 100% entirely clear on what the ‘all persons’ mean. Also, I mean I think the intention there is, is it only for patient-reported? And it would be better off to say that QOL assessments could be used with any respondent type. But I think that QOL can be person- or patient-reported, just they can be used with any respondent I didn’t like about this one. I just found that a little bit confusing.” GML#7 “So, this is quite health-centric. I would suggest that you could change your tagline to, ‘Together, let’s build a person-centred provincial system’ – something that leaves the opportunity for this to be available and utilized more broadly than just in health.” GML#9	Tweaked wording and graphics including added "data" to fact 1 after "patient reported"; Merged first 2 bullets into 1 bullet; Changed “groups” to “populations”; Made font size of "Quality of Life Assessments" larger and "Fact Sheet About" smaller in the title; Removed abbreviation from heading; Italicized introduction and bolded "older adults" and "family caregivers; Revised tagline for all documents to read “Together, let’s build a person-centred healthcare system for everyone.”
Brochure: “Frequently Asked Questions (FAQs) about QOL Assessments”		
“So, for me and this office, it’s a good introductory tool. Its concepts are good. It certainly highlights the key aspects of how this office looks at quality of life and looks at the opportunities for monitoring, measuring, and comparing quality of life in the long-term care sector. I could see that this office would make reference to these materials and conversations with other sectors of government – for example, Ministry of Finance or Ministry of Transportation, etc. Ministry of Health, they kind of already get this and understand this part. But other aspects of government that are not as involved with frailty issues, this is a nice primer that helps them understand what we mean by quality of life as an example and why it matters for seniors and how it can be advantageous.” GML#9	“Those who analyse and interpret these QOL assessment data **must** be experts in measurement. This [wording] may be a deterrent.” GML#7,8,9 and 10 “As a government leader, I’d be looking for the documents that informed this FAQ. So, I’d be wanting to see where the linkage is to the more fulsome documents that have informed the FAQ.” GML#9	Moved "What are QOL assessments" to introduction; Removed "person reported" under the “Improve Quality” section; Changed words “or their" to "and" and “over" to "across" under the top right fact; Changed the font size in the title; Removed abbreviation from heading; Italicized introduction and bolded "older adults" and "family caregivers"; Replaced “must” to “need” in the sentence “Those who analyse and interpret these QOL assessment data need to be experts in measurement”; Added the link to the peer-reviewed bibliography

All four participant groups		
Exemplar quotes by participants: overall responses to the resource	Exemplar quotes by participants: suggested revisions	Revisions made to the KT resource
Whiteboard video: “A Better Life: QOL Assessments”		
“Yes, her voice was succinct and clear., the general message was good. especially the ending. I liked the ending because say it’s… make it better for you. Yes, and to know that it is helpful and that there’s somebody working to try and make it easier for seniors as they approach a time of leaving this planet. It was everything that should be there. It was just the little bits of too fast. Other than that, it’s excellent.” PAC#2 “I think it got the general message across, again, as well – that’s improving quality of life and, briefly, why it’s so important. I think on the positive it was a short, short audio clip, which is good, I think it was clear enough.” HCP#8	“My parents are just average 80-year-old people. We need to tidy up the language and make it simple and straightforward so that people can follow… People who are going to be interested in this information are going to be people who are under stress. Either it’ll be a family caregiver who’s stressed and trying to understand frailty in regard to their own family, it needs to be simple and straightforward. it’s not warm and engaging sounding. The person has a great voice. So, the voice is good, but the language is not warm and engaging. It’s not about sort of a conversation with someone, even though the second person isn’t there.” GML#9 “Okay, first of all, it’s way too fast, way too fast. There’s a lot of words that have… You noticed I had my head turning? I don’t have a hearing aid in my left ear, but I do in my right ear. In my right ear, a lot of that was… I would’ve needed to read along with it to get everything. Even from my left ear, there were words that I knew what they were only because I could anticipate what was going to be said. Yeah, that is much too fast, and if possible, bring it down just a little bit.” PAC#14	Began video with an older adult living with frailty to make it more relational; Built QOL definition into the video; Improved the language including increasing frailty language and personalizing it through “our” and “we” language; Added a tagline "resource allocation" and changed tagline to "build a better healthcare system for everyone"; Decreased distracting qualities (for e.g., slowed down recording, muted colors and changed them to match other resources); Replaced house painting graphics with graphics suggesting preparing meals to make it more realistic
Video: “A Better Life”		
“So, I’m a very visual person, and I think the movie, video clip just captured all. It was very real. It didn’t feel staged. We see this all the time in the community, and it just sent that message out very strongly and clearly that this is something that’s happening more often than we actually realize – caregiver burnout, caregivers are underappreciated, they’re not compensated for this, and that there is that potential. So, yeah, very powerful and relational. I thought it had a significant impact. It was just very attention catching I think the video would be good specially to get the buy-in amongst other clinicians. So, whether that be in a rounds or a team meeting sort of setting, initiate, maybe, that the video run at the start and perhaps then working with them to try and get them on board with either participating in these questionnaires or running them to better capture.” HCP#8 “It’s a narrative, so it’s not just a lesson or a lecture, yeah. It was pretty human. Yeah. I wouldn’t hesitate to use it or steer people to it. Yeah, and the facial language was clear and easy to understand, too. [laughs]I thought it was interesting that they would choose a father and a son. I mean, it’s absolutely legitimate, so there’s no reason why they wouldn’t, but it certainly is probably not the most common.” PAC#15	“I assume the perky young lady is your doctor. We don’t know that for sure, but it looks like a doctor’s office and got the regulation stethoscope around her neck and so on. When the doctor was running through the list of the different kinds of areas that the QOL questionnaires cover, social wellbeing… Again, I think the average person on the street is going to say, ‘What?’” PAC#9 “I think it certainly gives you a hint, and as I said, it is only a short clip. But I wonder if sort of in that middle part if there couldn’t have been a little bit more explanatory… So, we looked at this and looked at this, and this is something that they seem to have in common and they both enjoyed, and so they opted to do this – you know, just that there was some thought process going on, not just you filled out this questionnaire and then we put it through some machine and came out and said, ‘This is what you need to do,’ right?” PAC#3 “I mean, it wasn’t offensive, but it wasn’t very realistic either. Because it just seemed so completely barren. Not even regular clutter, nothing like that. Just basically like a… like you just moved into a condo, but you didn’t even bring anything with you, yeah. Also, I’m thinking that maybe something more middle of the road, that there could be more discussion about oh, I didn’t realize that you were feeling so overburdened and that you needed a break, for example. Like, if there was some type of dialogue in it, that it might have been better.” HCP#10	Changed title from "A Good Life" to "A Better Life"; Streamlined the video to capture and hold the attention of the audience all the way through; Shortened the introduction montage (toothbrush sequence); Fixed lip-synch errors and removed fist bump; Color-corrected the kitchen scene; Ensured the video setting made clear that the “perky young lady” is a doctor by adding a clinic like surroundings; Provided more details about the process especially visuals showing father-son communicating and completing QOL assessment tools together

Looking across the groups, participants discussed potential benefits, perceived relevance, and usefulness of the KT resources. For example, one family caregiver appreciated the encouragement: *I* *like* *the* *repeating* *of* *the* *phrase* *‘living* *your* *best* *life* *possible,’* *because* *some* *days* *that* *doesn’t* *look* *very* *good* *for* *a* *lot* *of* *people.* Healthcare providers saw the linkage between completion of QOL assessment tools and follow-up discussions which may include the family. One explained: *It’s* *not* *focusing* *just* *on* *the* *client,* *but* *it’s* *also* *pulling* *in* *the* *family* *caregiver* *or* *the* *primary* *caregiver,* *which* *I* *think* *is* *really* *important* *if* *we’re* *going* *to* *be* *looking* *at* *a* *sort* *of* *patient-centred,* *family-centred* *care.* A healthcare leader perceived benefits of the KT resources at a meso-level: *Yes,* *I* *think* *it* *is* *speaking* *to* *us* *in* *the* *language* *that* *we* *understand* *around* *performance,* *accountability,* *all* *those* *things.* *I* *think* *that* *makes* *sense* *to* *me.* A government leader also perceived the value of QOL assessment data alongside other data used in decision-making: *I* *think* *you’re* *getting* *at* *kind* *of* *the* *critical* *ones* *[information],* *which* *is* *‘what* *is* *the* *value* *of-’* *or* *‘not* *the* *value* *of-’,* *and* *this* *kind* *of* *data* *‘holds* *up’* *next* *to* *administrative-level* *data* *or* *clinical-level* *data,* *which* *I* *think* *is* *very* *often* *kind* *of* *dismissed.*

Participants described what they liked about the KT resources, including ease of use, multiple modalities, and comprehensiveness. One family caregiver said, *It’s* *standard* *English,* *and* *there* *were* *no* *large* *words,* *‘onomatopoeia’* *or* *something* *like* *that!* Many participants across groups appreciated the multi-media approaches. A healthcare provider explained, *You’ve* *got* *a* *webpage,* *you’ve* *got* *some* *pamphlets,* *and* *you’ve* *got* *a* *variety* *of* *methods.* *And* *I* *think* *that’s* *really* *important,* *so* *I* *do* *really* *appreciate* *that.* *Because* *we* *know* *that* *when* *we’re* *trying* *to* *reach* *out* *and* *embed* *some* *new* *ways* *of* *working* *in* *our* *organization,* *that* *you* *need* *a* *lot* *of* *different* *resources…to* *support* *that.* *I* *appreciate* *the* *fact* *there’s* *sort* *of* *a* *multimodal* *approach.* Many participants commented on the simplicity of the messages. One government leader said, *Very* *clear,* *very* *crisp.* *I* *like* *the* *tagline,* *the* *mnemonic.* *I* *think* *having* *a* *fact* *sheet* *and* *a* *Q&A* *are* *pretty* *complementary,* *so* *I* *think* *that’s* *all* *there.*

Participants also offered recommendations for refinement. Older adults and family asked that the reading level be lowered and messages be simplified. As one older adult said, *I’m* *trying* *to* *put* *myself* *in* *the* *shoes…of* *somebody* *who* *has* *a* *Grade* *8* *reading* *level.* Some words/phrases like “enhance,” “healthcare team,” or “support system” were too academic, and were clarified. Both healthcare providers and leaders emphasized the need for references to evidence-based sources, and suggested that *less is more*, meaning that content be condensed so they could read/skim it quickly. One healthcare manager said, *I* *think* *they* *could* *be* *‘conciser’…what* *I* *find* *is* *the* *more* *that* *you* *go* *‘up’* *the* *organization,* *the* *simpler* *things* *have* *to* *get* *because* *the–what* *do* *you* *call* *it–the* *‘bandwidth’* *is* *very* *narrow.* Government leaders and decision-makers recommended various edits to avoid healthcare jargon and abstract language. One said, *Have* *one* *more* *layer* *on* *‘how.’* *It’s* *still* *a* *bit* *theoretical,* *still* *a* *bit* *academic.* A few participants found the layout or colors not appealing, visuals not sufficient, or the font size too small. Participants suggested revisions to the whiteboard and live-action video. Refinements included slowing down the pace of both videos, adding tag lines or images to help with flow, and reducing the length. The KT resources were subsequently refined based on the formative feedback and final versions were made available to all participants and freely online at www.healthyqol.com/older-adults.

## Discussion

This study was motivated by a need for KT resources that addressed knowledge gaps of micro-, meso-, and macro-audiences regarding the use of QOL assessment tools for older adults and family caregivers. Our results confirmed the need for introductory resources, and the importance of tailoring these resources to specifically address the knowledge gaps of different audiences. Older adults and family caregivers wanted to understand how their QOL information could be collected, reported, and used to improve their care. Healthcare providers emphasized the need for practical information on how to integrate and use QOL assessment tools in their practice. Healthcare managers and leaders focused on information about using standardized tools, like PROMs and PREMs, for care decisions and quality improvement in their organizations. Government leaders and decision-makers required evidence-based information on using PROMs and PREMs for macro-level purposes to monitor performance, improve quality of care, and make budget decision in healthcare systems. Our project serves as an exemplar of how such information can be used to develop introductory KT resources tailored for different audiences.

There are various other evidence-based resources available to support the use of QOL assessment tools in healthcare [28–33]. Notable examples include a user’s guide on patient-reported outcomes in clinical practice by the International Society of Quality of Life Research [28, 46, 47], and guidelines for the use of electronic patient-reported outcomes [32, 48]. Additional guidance is provided by a range of theories and systematic reviews on QOL assessment [8, 14, 15, 49–52]. These resources provide invaluable, detailed, evidence-based information for healthcare providers and organizations motivated to use QOL assessments. Our previous work [26, 27, 53] identified that for those user groups who were not yet convinced of their importance, there was a need to develop KT resources to *introduce* QOL assessment and “what’s in it for me?” For QOL assessment tools to be used, it is important that users first have a basic understanding of their value and potential use for different purposes. Our project addressed this need by specifically focusing on the development of tailored introductory resources through engagement with patients, clinicians, and decision-makers.

In addition to developing tailored content, it is also important to develop resources that use language and modality that is familiar, supportive, and accessible to the different audiences. To achieve this, working with a language specialist and graphic designer may be required. With respect to language, it is important for reading levels to match the audience. For example, for patients and family caregivers, a Grade 6 reading level (or below) is generally recommended [54–56]. In addition, different audiences are familiar with different terminology, designs, and formats. For example, healthcare leaders and decision-makers may not be familiar with the term “QOL assessment” and more commonly use PROMs and PREMs, whereas the terms “QOL” and “assessment” may be more familiar to patients, family caregivers, and clinicians. Government leaders may be more familiar with short briefing notes, whereas conventional continuing education formats (e.g., manuscripts) are more familiar to clinicians. Accessibility to patients and family caregivers could be enhanced by providing materials both in written and audiovisual formats.

While our work was guided by a well-established KT framework [34, 35], there are limitations. First, we had limited diversity among participants, thus various ethnic groups, geographies (e.g., remote), and living arrangements (e.g., multigenerational households and structure) may not be sufficiently attended to in our KT resources. Second, we did not fully address the question of *how* to develop KT resources. The current project may serve as an exemplar for a further project in this area with specific emphasis on further tailoring of resources for patients, family caregivers, and healthcare providers with diverse backgrounds and other healthcare systems. In so doing, we particularly recommend studies in different languages, healthcare systems, and diverse populations. Third, our work did not entail summative evaluation for end-of-project evaluation of the KT resources, which may be taken up in future work.

## Conclusion

Through this study, we offer three important contributions to the field. First, our findings identify that knowledge gaps of micro-, meso-, and macro-audiences regarding QOL assessment are distinct and their needs must be addressed. Second, our results affirm the need to tailor evidence-based KT resources to address knowledge gaps that may hinder different audiences’ use of QOL assessment tools. Third, while evidence-based information is available for those already motivated to use QOL assessment tools, through an integrative KT approach we produced tailored, introductory KT resources for those who may still be asking: “what’s in it for me?” Our study exemplifies engaging patients, family caregivers, clinicians, and decision-makers in developing such resources to address their unique knowledge gaps and support the use of QOL assessment tools for older adults and family caregivers.

## Data Availability

Ethical approval was not attained for the purposes of public sharing of the data. Scientists who are interested in using the data may contact the corresponding author for further information.
